# Influence of patient-related characteristics on early migration in calcar-guided short-stem total hip arthroplasty: a 2-year migration analysis using EBRA-FCA

**DOI:** 10.1186/s13018-016-0363-4

**Published:** 2016-03-07

**Authors:** Karl Philipp Kutzner, Mark Predrag Kovacevic, Tobias Freitag, Andreas Fuchs, Heiko Reichel, Ralf Bieger

**Affiliations:** Department of Orthopaedic Surgery and Traumatology, St. Josefs Hospital Wiesbaden, Beethovenstr. 20, 65189 Wiesbaden, Germany; Clinic of Traumatology, Hand- and Orthopaedic Surgery, HELIOS Dr. Horst Schmidt Clinic Wiesbaden, Ludwig-Erhard-Str. 100, 65199 Wiesbaden, Germany; Department of Orthopaedic Surgery, University of Ulm, Oberer Eselsberg 45, 89081 Ulm, Germany

**Keywords:** Total hip arthroplasty, Migration, Subsidence, Short stem, optimys, EBRA, Weight, BMI

## Abstract

**Background:**

Short stems have gained popularity in recent years. Because of encouraging clinical results, indications have been expended from young to elderly and obese patients. However, long-term results are lacking. The purpose of this study was to evaluate the influence of gender, age, body weight, body mass index (BMI), and offset version on short-stem migration in correlation to the clinical outcome.

**Methods:**

The implant migration of 202 metaphyseal-anchoring, calcar-guided short stems in 151 patients was assessed by “Einzel-Bild-Roentgen-Analyse” femoral component analysis (EBRA-FCA, femoral component analysis) in a 2-year follow-up. Full weight bearing was allowed directly after surgery. Patients were divided into groups regarding gender, age, body weight, BMI, and offset version. The Harris hip score (HHS) and satisfaction on visual analogue scale (VAS) were analyzed.

**Results:**

After 2 years, mean axial subsidence of all 202 implants was 1.43 mm (standard deviation, SD 1.45 mm). A continuous reduction of initially pronounced subsidence over time could be observed. None of the stems had to be revised. Statistically significant increased rates of subsidence were seen in male (1.68 mm; SD 1.56 mm; *p* = 0.005) and heavy patients (1.54 mm; SD 1.48 mm; *p* = 0.022). No differences in implant migration were found regarding age, BMI, and different offset versions. HHS improved markedly from 45.8 (SD 15.9) to 98.1 (SD 4.7) while satisfaction on VAS improved from 1.8 (SD 2.2) to 9.7 (SD 0.9) after 2 years.

**Conclusions:**

The results suggest a migration pattern with initially pronounced subsidence followed by subsequent stabilization. Male and obese patients show a slightly increased initial subsidence without any signs of sustained micromovement. No correlation was found concerning clinical results and pronounced initial subsidence above the threshold of 1.5 mm. No aseptic loosening or other signs of implant failure were seen within the observation period of 2 years.

**Trial registration:**

German Clinical Trials Register, DRKS00009834.

## Background

Cementless short stems have gained popularity in total hip arthroplasty (THA) in recent years [1–3]. A broad variety of different designs emerged on the market, proposing a more physiological load transfer while offering the possibility of less invasive surgery, preserving femoral bone stock, and sparing soft tissue [4–7]. Recent studies proved beneficial mid-term clinical results compared to conventional straight stems with decreased intraoperative complication rates [8–10]. These encouraging results led to the expansion of indications of short stems, including young and more active patients, elderly patients with reduced bone quality, and obese patients [11–13]. In addition, simultaneous bilateral implantation of a metaphyseal-anchoring short stem resulted in excellent clinical and satisfying radiological outcomes in the early stage [14]. However, to date, clinical and radiological long-term results are still lacking.

The most common reason for implant failure is aseptic loosening [15]. In this context, primary stability, as well as the design-specific potential to maintain proximal femoral bone stock, is an important feature in order to predict implant survival [16]. In short-stem THA, a major concern in reducing diaphyseal fixation of the femoral stem is still the possible reduction of implant stability and the increase of interface micromotion which, by interfering osteointegration, might increase the risk of implant loosening [16]. Early implant migration in conventional THA was found to be the best predictor of mechanical failure and represents an important factor impairing the long-term survival [15, 17, 18]. “Einzel-Bild-Roentgen-Analyse” (EBRA) measurements showed that axial subsidence of more than 1.5 mm after 2 years in conventional cementless straight-stem THA was predictive for late aseptic loosening and a possible increase in the risk of revision [18]. To date, it is unknown if this prediction can be transferred to short-stem THA as well.

Recently, several short-stem designs showed comparable primary stability to conventional straight stems in in vitro studies [16]. However, only a few in vivo studies analyzing the migration pattern of short-stem implants using either EBRA femoral component analysis (EBRA-FCA) or Roentgen-stereophotogrammetry (RSA) technique have been published [10, 19–22]. Regarding the risk of aseptic loosening, case control studies have indicated a multifactorial etiology including not only implant design but also patient-related factors, such as gender, age, weight, height, and body mass index (BMI) [23, 24]. To date, little is known about the influences of patient-related factors on stem migration as a potential reason for revision [20, 25].

The objective of the present EBRA-FCA analysis was to assess the influence of patient-related factors such as gender, age, weight, BMI, and different offset versions on the migration pattern of a metaphyseal-anchoring, calcar-guided short stem in a 2-year follow-up. Additionally, the clinical outcome was analyzed in order to determine differences in those cases with pronounced subsidence.

## Methods

After institutional review board approval (University of Ulm, Germany, 323/13), 216 consecutive short-stem implantations in 162 patients could be retrospectively included at a single institution from an ongoing prospective observational study. Written consent to participate has been obtained from all patients. Between 2010 and 2012, 108 patients underwent unilateral THA, and in 54 patients (108 hips), simultaneous bilateral THA was performed. The indications for implantation were as follows: 91.7 % (*n* = 198) primary osteoarthritis, 5.1 % (*n* = 11) femoral head necrosis, 2.3 % (*n* = 5) congenital dysplasia, and 0.9 % (*n* = 2) secondary osteoarthritis. The following inclusion criteria were applied: a minimum follow-up of 2 years, a series of at least three consecutive standardized radiographs accepted by the EBRA-FCA software, and acceptance of the direct postoperative and the 24-month follow-up radiograph.

All patients underwent pre- and postoperative digital anteroposterior imaging using a standardized technique. A positioning splint with 20° internal rotation of hip joints was used in order to achieve a standardized and reproducible image during follow-up. The X-ray tube was positioned at a 1-m distance to the table in the perpendicular position. The follow-up included a maximum of five postoperative radiographs: during hospital stay, 6 weeks, 6 months, 12 months, and 24 months after surgery.

Mean follow-up time was 2.2 years (range 2.0–3.0 years). Ten hips had to be excluded because of an incomplete radiological series and three patients (four hips) deceased unrelated to the operation with prosthesis in situ. In total, 202 hips in 66 female and 85 male patients fulfilled the inclusion criteria, resulting in a follow-up rate of almost 94 % (Table 1).

**Table 1:** Patients’ characteristics

		Patients’ characteristics			
Gender		Age	Weight	Height	BMI
Females	*n*	66	66	66	66
	Mean (SD)	62.8 (9.28)	77.4 (18.28)	166.6 (5.29)	27.7 (6.01)
	95 % CI	60.5, 65.0	72.9, 81.9	165.3, 167.9	26.3, 29.2
	Median	63.2	74.0	167.5	26.0
	Range	33–87	50–140	152–178	19–45
Males	*n*	85	85	85	85
	Mean (SD)	63.3 (9.84)	89.0 (15.10)	177.7 (6.64)	28.2 (4.53)
	95 % CI	61.2, 65.4	85.8, 92.3	176.3, 179.2	27.2, 29.2
	Median	62.9	84.0	178.0	27.0
	Range	37–81	66–153	165–192	22–43
Total	*n*	151	151	151	151
	Mean (SD)	63.1 (9.58)	83.9 (17.50)	172.9 (8.21)	28.0 (5.21)
	95 % CI	61.5, 64.6	81.1, 86.7	171.5, 174.2	27.1, 28.8
	Median	62.9	82.0	172.0	27.0
	Range	33–87	50–153	152–192	19–45

*n* number of cases, *SD* standard deviation, *CI* confidence interval

**Fig. 1:**
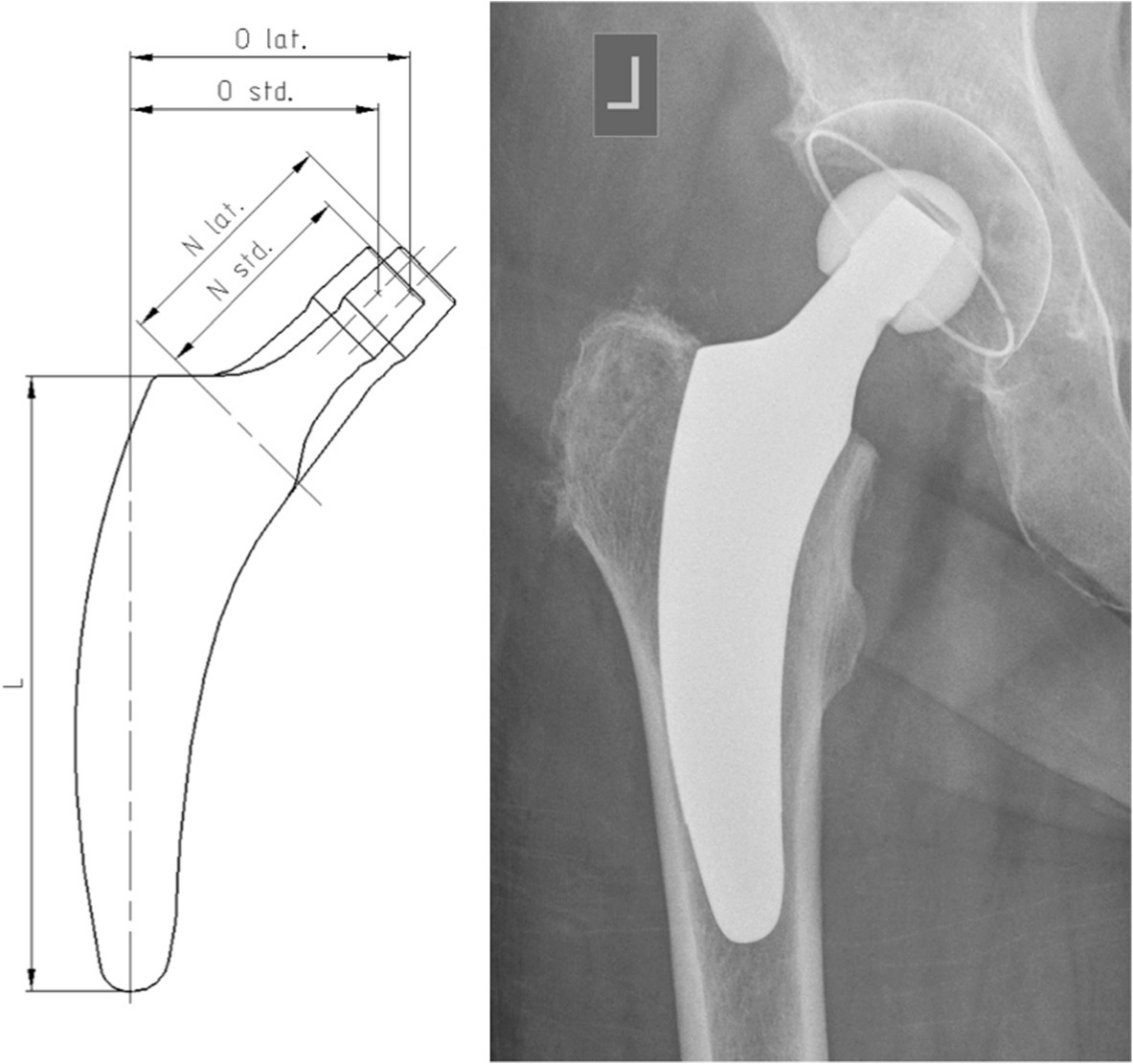
The optimys stem offers two different offset versions (standard/lateral)

All patients received a cementless, calcar-guided short stem (optimys®, Mathys Ltd, Bettlach, Switzerland) (Fig. 1). The optimys short stem is a type 2A short stem according to the classification of Khanuja et al. [26]. It is made of a titanium alloy with a plasma-sprayed surface and a calcium phosphate coating. The profile of the stem is tapered in three planes with a trapezoidal cross section to provide femoral press-fit fixation. The implant is aligned along the proximal medial cortex and the calcar femorale. Anchoring is based on the fit-and-fill principle but can also be done as the classic three-point anchoring in some cases. The greater trochanter region remains intact. There are two different offset options (standard and lateral offset) to optimally reconstruct the individual anatomy (Fig. 1). The stem was combined with a cementless press-fit cup (RM Pressfit vitamys, Mathys Ltd Bettlach, Switzerland or Fitmore cup, Zimmer, Warsaw, USA) with a 28-mm alumina-on-highly crosslinked polyethylene bearing in all hips. All surgeries were performed in supine position using a modified, minimally invasive anterolateral approach [27]. Full weight bearing using two crutches was allowed in all cases immediately after surgery.

EBRA-FCA was used to determine axial subsidence of the stem [28]. Therefore, the images were calibrated using the diameter of the prosthetic head. A total of 19 reference points were defined on the femoral head (6), the stem (3), the femoral cortex (8), and one at the greater and lesser trochanter. These reference points define predetermined distances, which are compared by the EBRA-FCA software to calculate implant migration. Radiographs with significant positioning artifacts were excluded by the EBRA-FCA software.

The influence of patient-related factors such as gender, age, weight, and BMI was evaluated in comparison to migration of the whole group. Therefore, patients were divided into the following groups: male versus female, < 65 versus > 65 years (resulting in two equally sized groups), < 75 versus > 75 kg (according to previous literature [25]), < BMI 30 versus > BMI 30, and offset version standard versus lateral.

To correlate migration measurements to clinical results, the Harris hip score (HHS) and satisfaction on visual analogue scale (VAS) were assessed during follow-up. In one patient, clinical follow-up could not be performed after 2 years.

For statistical evaluation of subsidence, the last follow-up record was used. Differences between groups were examined non-parametrically using Wilcoxon two-sample tests and Kruskal-Wallis test (i.e., in case of more than two groups). To break up the effects of gender and weight, an analysis of variance model was also carried out incorporating gender and weight and including age as predictors. Least squares means were calculated as estimates of the adjusted means. For statistical significance, a *p* value of less than 0.05 was considered. The SAS software 9.4 was used for all analyses (SAS Institute, Cary, NC, USA).

## Results

A total of 954 radiographs were analyzed by one observer. The EBRA-FCA software accepted 942 images and rejected 12 radiographs (1.2 %). A continuous reduction of axial subsidence over time could be observed. After 6 weeks, mean axial subsidence was 0.55 mm (standard deviation, SD 0.78 mm); after 6, 12, and 24 months, it resulted in 0.90 mm (SD 1.02 mm), 1.14 mm (SD 1.18 mm), and 1.43 mm (SD 1.45 mm), respectively. Eighty of 202 (39.6 %) implants showed axial subsidence of 1.5 mm or more after 24 months.

**Fig. 2:**
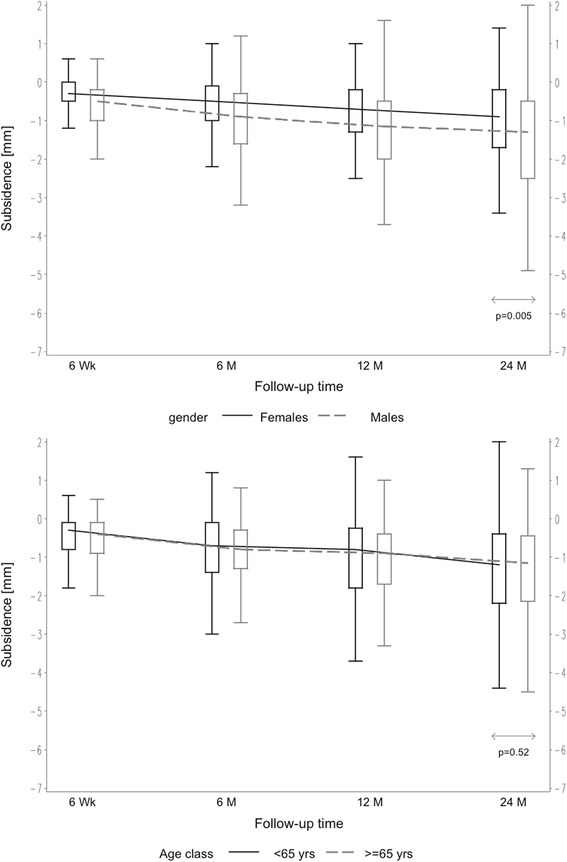
Box plots of axial subsidence by patient-related criteria (gender and age)

**Fig. 3:**
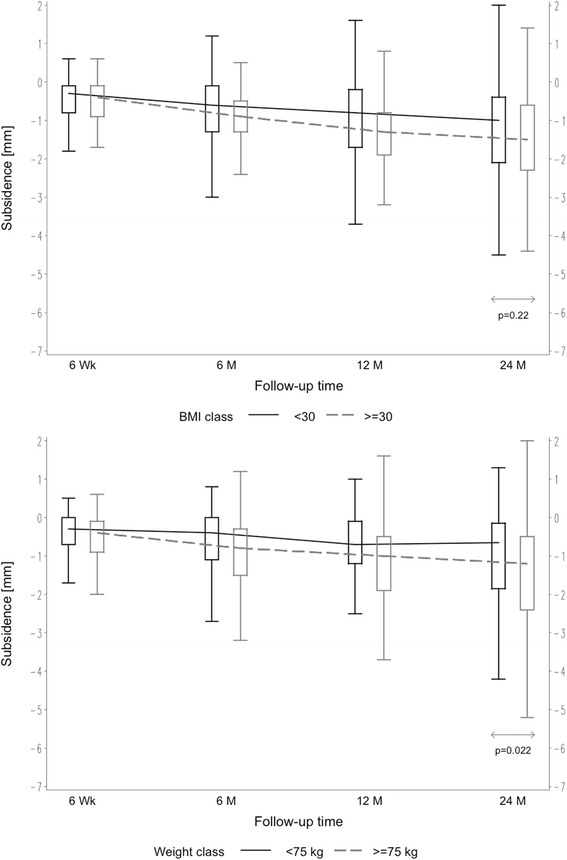
Box plots of axial subsidence by patient-related criteria (weight and BMI)

**Fig. 4:**
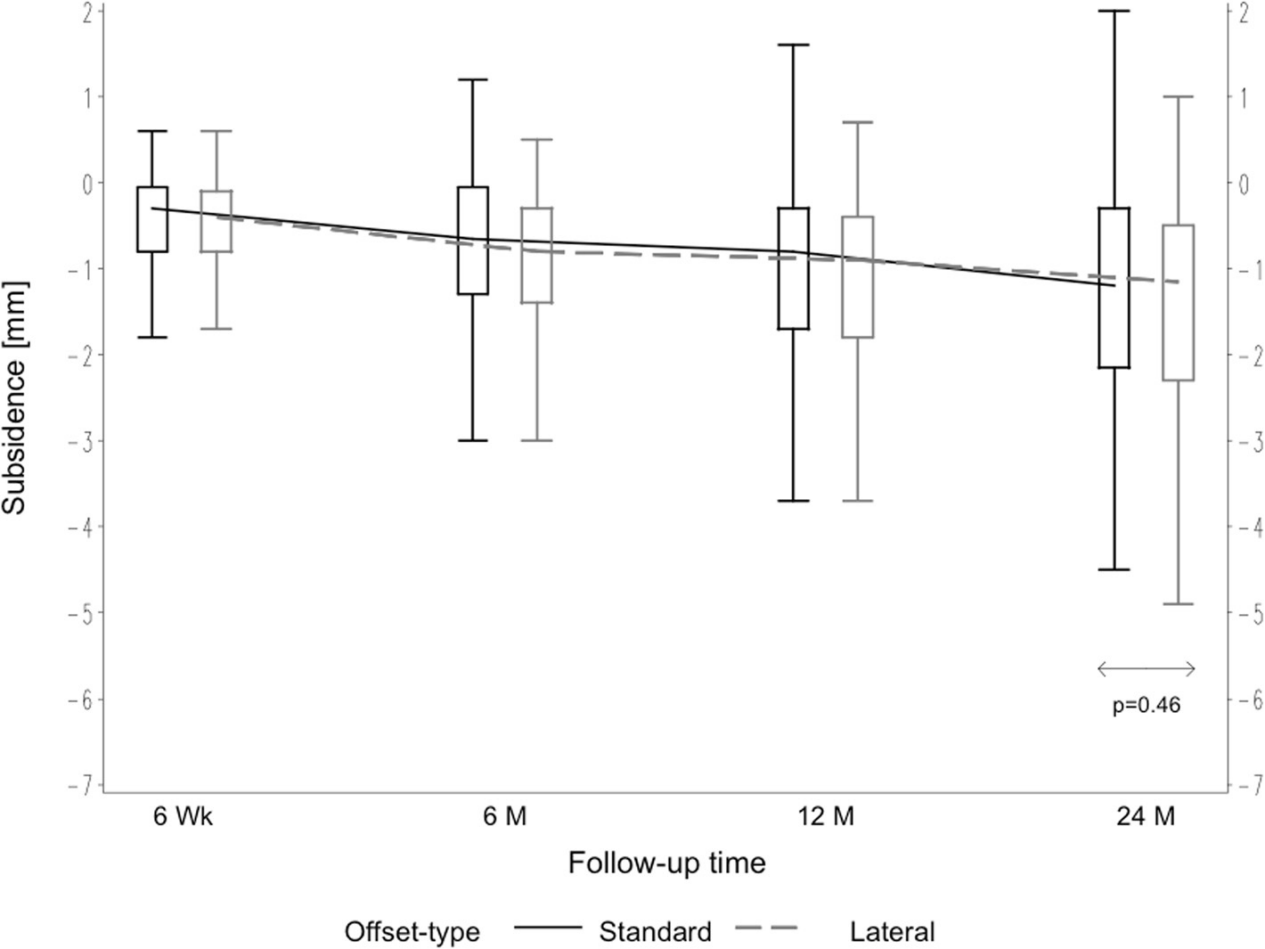
Box plots of axial subsidence by different offset versions (standard/lateral)

Patients were divided into groups in order to evaluate patient-related factors on axial stem subsidence. Comparing male versus female patients, mean axial subsidence was 1.68 mm (SD 1.56 mm) in males versus 1.09 mm (SD 1.21 mm) in females after 24 months (*p* = 0.005) (Fig. 2). A significant influence of weight on mean axial subsidence was seen. Comparing weight groups of < 75 versus > 75 kg, subsidence was measured to be 1.09 mm (SD 1.30 mm) versus 1.54 mm (SD 1.48 mm), respectively, after 24 months (*p* = 0.022) (Fig. 3). However, based on the results of the analysis of variance, the age- and gender-adjusted least squares mean subsidence for weight < 75 kg was 1.18 mm and 1.47 mm for weight ≥ 75 kg, respectively. Weight itself did not appear to be significant when adjusting for age and gender. Likewise, the age- and weight-adjusted mean subsidence for gender was attenuated in the model: the least squares mean of females amounting to 1.09 mm and 1.56 mm for males but still being significant (*p* = 0.036). But the model accounts for 6.3 % of the total variation only; hence, the predictive value is somehow limited. In turn, different BMI showed no influence on the amount of axial subsidence. Groups of BMI < 30 versus > 30 kg/m^2^ resulted, respectively, in mean subsidence of 1.38 mm (SD 1.45 mm) versus 1.56 mm (SD 1.45 mm) (*p* = 0.22) (Fig. 3). There was no effect seen regarding different age groups (< 65 versus > 65 years), with mean axial subsidence of 1.42 mm (SD 1.48 mm) versus 1.43 mm (SD 1.42 mm), respectively, after 24 months (*p* = 0.52) (Fig. 2). The distribution of the two different offset versions (standard and lateral offset; Fig. 1) was rather uniform. In 100 cases (49.5 %), a standard offset stem was implanted, while in 102 cases (50.5 %), a lateral offset version was used. Two years after surgery, there was no statistical difference in mean axial subsidence in reference to the stem type used (standard 1.37 mm (SD 1.52 mm) versus lateral 1.49 mm (SD 1.37 mm)) (*p* = 0.46) (Fig. 4).

HHS improved from 45.8 (SD 15.9) preoperatively to 98.1 (SD 4.7) 2 years after surgery while satisfaction on VAS improved significantly from 1.8 (SD 2.2) preoperatively to 9.7 (SD 0.9) after 2 years. There was no evidence of a difference in HHS (*p* = 0.764) and satisfaction on VAS (*p* = 0.785) in the group of patients with measured subsidence of > 1.5 mm compared to the group of subsidence of ≤ 1.5 mm (Table 2). After 2 years, none of the stems had to be revised and there was no evidence for aseptic loosening or implant failure.

**Table 2:** Clinical outcome after 2 years comparing axial subsidence ≤ 1.5 and > 1.5 mm

		Clinical outcome	
Subsidence		HHS (*p* = 0.764)	VAS satisfaction (*p* = 0.785)
≤ 1.5 mm	*n*	122	122
	Mean (SD)	98.0 (5.05)	9.7 (1.02)
	95 % CI	97.1, 98.9	9.5, 9.9
	Median	100.0	10.0
	Range	65–100	2–10
> 1.5 mm	*n*	79	79
	Mean (SD)	98.4 (3.66)	9.7 (0.63)
	95 % CI	97.6, 99.2	9.6, 9.9
	Median	100.0	10.0
	Range	80–100	7–10

Values of one case in the > 1.5-mm group are missing

*n* number of cases, *SD* standard deviation, *CI* confidence interval

## Discussion

The present study analyzed the migration pattern of a metaphyseal-anchoring, calcar-guided short stem in a 2-year follow-up and the influence of patient-related factors on early subsidence. After an initially pronounced subsidence, stabilization can be observed over time. While no impact was found for age, BMI, and offset versions, increased rates of subsidence were seen in heavy and male patients. However, apparently weight is confounded by gender with females having much less weight as compared to males.

There are only a few studies analyzing early migration pattern of cementless stems in association with patient-dependent factors. The increased rate of subsidence has been related to osteoporotic bone [29], heavy patients [25], and male patients [30] using conventional cementless stems. However, to date, it remains unclear if the increased rate of subsidence in men is related to factors of weight or activity levels [30]. Stihsen et al. [25] in an EBRA-FCA analysis found that body weight over 75 kg significantly impacts on subsidence and the stability of the vision 2000 stem (Depuy, Warsaw, Indiana). After 2 years, mean subsidence of 102 implants was 1.38 mm. Freitag et al. [20] reported a tendency towards increased subsidence in patients with a BMI > 30 kg/m^2^ analyzing 72 cases after Fitmore stem implantation (Zimmer, Warsaw, Indiana) without finding statistical differences. In a recent investigation, Kaipel et al. [10] found no influencing factors such as gender, age, BMI, or implant size on the initial vertical migration of the Nanos stem (OHST Medizintechnik AG, distributed by Smith & Nephew, Marl, Germany). However, the number of patients included might not allow valid analyses [10]. In the present study, an increased rate of early subsidence was found in patients with body weight over 75 kg, suggesting a cautious confirmation of indication in heavy patients. The operating surgeon should be aware that high body weight may lead to increased early stem migration. However, the critical threshold of early implant migration remains unclear. Interestingly, BMI > 30 kg/m^2^ did not influence the rate of early subsidence in our investigation, which confirms earlier studies [25]. The effect of gender, found in the present investigation, can be considered to be mainly affected by the different weight distributions of male and female patients. Besides patient-related factors, surgical technique might influence stabilization into the femoral bone, especially in heavy patients. Stems providing a poor fit and fill into the bone with lack of cortical contact show significantly higher odds of migration compared to those with a tight fit [25].

Most importantly, the rate of subsidence is associated with the stem design [21, 31, 32]. Not only the design of newly developed short stems but also the type of anchorage into the bone differs from conventional straight-stem designs. While fully coated conventional stems offer a diaphyseal anchorage, proximally coated stems have been developed to reduce diaphyseal anchorage and increase metaphyseal fixation in order to avoid stress shielding [33]. The newest generation of short stems aims at a physiological metaphyseal fixation and load transmission [34]. However, even small disparities in stem design might lead to changing the biomechanical properties [32]. Consequently, this might result in a different migration pattern of metaphyseal-anchoring short stems compared to conventional stem designs. Diaphyseal anchoring and especially distally coated implants reach stability by intraoperatively achieving a fit and fill in the diaphysis with tight cortical contact. However, given the distal anchorage and a non-physiological distal load transfer, vibrancy and micromovement might occur in the proximal part over time possibly resulting in deferred continuous migration [35, 36]. In contrast to the initial stability followed by secondary subsidence, several metaphyseal-anchoring short stems have been shown to present with an emphasized early subsidence followed by secondary stabilization [10, 19, 20, 37]. The stabilization pattern of a short stem can be explained by the curved and tapered shape of the stem design, which leads to a wedging in the proximal part of the femur accompanied with the impaction of trabecular bone, as it has already been described for the Mayo short stem [38]. In most cases, diaphyseal anchorage is not pronounced. The clinical relevance of early settlement within the proximal femoral bone followed by stabilization explicitly given the biomechanics of a calcar-guided short stem to date is not fully understood. A retrospective EBRA-FCA study in a 10-year follow-up using conventional stems showed that initial subsidence with secondary stabilization can succeed and is not inevitably followed by implant failure [18]. Revision for aseptic loosening was more common in patients with late-onset subsidence and in patients with early-onset followed by continuous further subsidence. These results suggest that the threshold of 1.5 mm for the rate of axial stem migration in the prediction of aseptic loosening and implant failure, which has been postulated in conventional stems [18], might not be valid for the design of calcar-guided short stems. Future migration analyses after 5 years using EBRA-FCA will show if the attainment of a stable state can be confirmed. Further periodic monitoring of patients particularly with pronounced initial subsidence is mandatory in order to detect signs of loosening and failure. In a study of Freitag et al. [20], 18 of 72 (25 %) Fitmore stems subsided more than 1.5 mm in the first 2 years without any implant revision in the observation period. However, this trochanter-sparing implant only partially anchors in the metaphysis and substantially differs from modern short-stem designs [10]. The investigated short stem can be classified as type 2A according to Khanuja et al. [26]. It was found that after a mean observation period of 2.9 years the cumulated short-term survival of type 2A short stems results in over 98 % [26]. In the present study, none of the stems had to be revised and no signs of aseptic loosening or implant failure were obvious. A continuous reduction of initially pronounced subsidence over time could be observed suggesting a stable state after 2 years. The results of HHS and satisfaction on VAS after 2 years prove excellent clinical function. No correlation was found concerning clinical results and pronounced initial subsidence above the threshold of 1.5 mm (Table 2).

The present investigation has several limitations. First to be mentioned is the short follow-up of 2 years. However, the migration pattern after 2 years has been established in several studies providing a reference to long-term survival [18, 20]. Furthermore, RSA provides higher accuracy in comparison to the EBRA-FCA method used in the present study. The computer-assisted EBRA-FCA system was evaluated to be able to detect stem subsidence of ±1 mm and varus/valgus tilting of ±0.4° given a specificity of 100 % and sensitivity of 78 % [28]. Nevertheless, the need to implant markers intraoperatively restricts the usage of RSA significantly and would have caused intense cost and effort given the large group of patients included in this objective. Due to the size, the reliability of the study group is considerable and we are confident of accurate results. However, an RSA analysis should confirm the findings in the future. In addition, in the present study, only axial subsidence was analyzed. Stem tilting and rotation were not subject to the investigation.

## Conclusions

The migration pattern of the investigated calcar-guided short stem can be characterized as an initially pronounced subsidence in order to settle-in within the trabecular metaphyseal bone followed by subsequent stabilization over time. Increased initial subsidence was seen in male and obese patients, indicating a cautious procedure in those cases. However, no signs of sustained micromovement could be observed. No correlation was found concerning pronounced initial subsidence and clinical results. Clinical results are very encouraging. No aseptic loosening or other signs of implant failure were seen after 2 years. The threshold of 1.5 mm for the rate of subsidence might not be valid for the design of calcar-guided short stems, but further monitoring is mandatory.

## Abbreviations

BMI: body mass index
CI: confidence interval
EBRA-FCA: “Einzel-Bild-Roentgen-Analyse” femoral component analysis
HHS: Harris hip score
RSA: Roentgen-stereophotogrammetry
SD: standard deviation
THA: total hip arthroplasty
VAS: visual analogue scale
